# Severe lupus nephritis in the present days

**DOI:** 10.3389/fneph.2022.984613

**Published:** 2022-08-24

**Authors:** Gabriella Moroni, Marta Calatroni, Claudio Ponticelli

**Affiliations:** ^1^ Department of Biomedical Sciences, Humanitas University, Pieve Emanuele Milan, Italy; ^2^ Nephrology and Dialysis Division, Istituto di Ricovero e Cura a Carattere Scientifico (IRCCS) Humanitas Research Hospital, Milan, Italy; ^3^ Independent Researcher, Milan, Italy

**Keywords:** severe lupus nephritis, systemic lupus erythematosus, immunosuppression therapy, kidney biopsy, renal insufficiency

## Abstract

Lupus nephritis (LN) is one of the most frequent and severe organ manifestations of systemic lupus erythematosus (SLE) that is a chronic autoimmune disease. Despite improvement in patient and renal prognosis, the disease continued to be associated with a high rate of end stage kidney disease. Along the last decades, it seems that the epidemiology of LN and its clinical presentation have progressively changed. The forms with renal insufficiency at presentation seem to have progressively reduced in developed countries in favour of more mild clinical presentations with urinary abnormalities only. To this clinical change does not correspond a less severe histological lesions, in fact, the extent of active lesions at kidney biopsy are unchanged, whereas chronic lesions are becoming less frequent and less severe. Meanwhile, new types of severe LN defined by the variable association of demographic, clinical, histological characteristics at diagnosis or during the follow-up are gradually emerging and require attention in assessing the therapy and prognosis.

During the last years, randomized controlled trials have reported the efficacy of new drugs in association with standard therapy to improve the rate of short- and medium-term renal response. One of the advantages is that these results were obtained with reduced dosage of corticosteroids whose protracted use is associated with increase of chronic organ damage. Optimization of therapeutical strategies, tailored on the demographic clinical and histological characteristics, with combination of old and new drugs are urgently needed for severe LN.

## Introduction

Despite progressive improvement in renal prognosis (1, 2), proliferative lupus nephritis (LN) is still associated with a 6-fold increase in mortality compared with the general population (3). Around 10% of patients develop end-stage kidney disease (ESKD) within 5 years from the diagnosis (4). Therefore, a prompt clinical and histological identification of LN patients at risk of progression and appropriate treatment in the initial and maintenance phases of the disease are critical to improve the outcome of patients with severe forms of LN. In this paper, we will review the clinical and therapeutic approaches to “severe LN”, mainly based on the last European League Against Rheumatism and European Renal Association-European Dialysis and Transplant Association (EULAR/EDTA) recommendations (5) and recent randomized controlled trials.

## What is severe lupus nephritis?

The term severe LN may be used to indicate the absent or the incomplete response to first-line conventional therapy with corticosteroids and immunosuppressive agents. However, LN can also be defined “severe” in the presence of clinical, histological, and/or demographic features that can predict a poor outcome, at diagnosis or during the disease course.

Clinically, increased levels of serum creatinine, high grade proteinuria, and active urine sediment are important prognostic signs of progressive and severe forms of LN (6). The occurrence of renal flares along the course of the disease is another predictor of poor kidney prognosis (7, 8).

Histologically, six classes of glomerular lesions have been defined in LN at kidney biopsy (9). Of them, class III (focal proliferative lupus nephritis), class IV (diffuse proliferative LN), and class III or IV plus class V are considered the most severe forms (10). Chronicity index can further contribute to define the possible outcome. The presence of tubulo-interstitial injury, glomerular sclerosis or fibrous crescents at basal kidney biopsy is a strong predictor of kidney function impairment (11, 12).

Apart from glomerular classification, other rare histologic forms may lead to severe LN, including vascular lesions in patients with antiphospholipid syndrome and thrombotic microangiopathy (TMA). In patients with antiphospholipid antibodies (aPL), glomerular and vascular lesions over-imposed to those of LN can be present and may lead to severe acute kidney injury (AKI) or chronic irreversible lesions (13–15). However, in a recent prospective cohort of 64 patients with biopsy-proven LN, aPL was associated with renal dysfunction in the short-term but had no deleterious effect on long-term renal survival (16). TMA is characterized clinically by thrombocytopenia and microangiopathic hemolytic anemia. It is an uncommon pathological finding in LN, with a prevalence below 10% (17). The characteristic vascular lesions consist in endothelial cells swelling with narrowing of vascular lumen and formation of thrombi. Renal TMA in LN is more frequent in patients with aPL antibodies, thrombotic thrombocytopenic purpura, malignant hypertension, or presence of anti-Ro antibodies (18). Complement activation by classical or alternative pathways plays a key role in the pathogenesis of secondary TMA (19). Whatever the cause, TMA in LN is associated with severe clinical presentation and bad long-term renal prognosis (20).

Among the demographic characteristics male gender, young age, non-Caucasian/Asian ethnicity, and poverty may be associated with more frequent and severe LN. Males account for 4% to 22% of SLE patients, with 30% in studies on familial aggregation (21). The incidence of males with LN seems to be increased in the last decades (2). Some investigators reported that males have more severe disease, showing more frequent clinical presentation with nephrotic syndrome or renal dysfunction, higher renal activity index at kidney biopsy, and more frequent progression to renal failure (22–26). Other studies demonstrated that appropriate therapy allows a benign course in males with LN (27). A review of 16 studies, reported that 6 studies pointed to an increase in incidence of LN in males, 9 studies demonstrated no disparity in gender, and one study showed contradicting results. In addition, 4 studies pointed that male had a more severe renal outcome as revealed by laboratory tests. However, the risk of dialysis and remission were similar between both genders. Young age is another predisposing factor of LN development in SLE (4) and it is associated with more severe presentation and outcome (28–30). In comparison with adults, juvenile-onset SLE has more frequently severe clinical manifestations of LN, a higher risk of flares, organ damage and higher mortality rates (31, 32). A poor adherence to therapy is frequent in children and adolescents with LN and can contribute to unpredictable flares and high levels of morbidity and mortality (33, 34). Ethnicity has a relative impact on the outcome of the patients and their response to treatment, but it needs to be taken into consideration in treatment decisions. SLE is more common and it is associated with a higher level of disease activity and poorer outcomes in Black, Hispanic and Asian patients (35). The severe lupus phenotype in those populations can be explained by increased autoantibody reactivity, higher frequency of arterial hypertension, and the genetic risk burden. Ethnicity remains a key determinant of poor SLE outcome, including, flares, ESKD and mortality, even after adjustment for socio-economic factors (36, 37). Low socio-economic status and poverty are associated with frequent flares, poor quality of life and increased morbidity and mortality in patients with SLE (38–40). Altogether, young age at onset(<30years), male sex, African-American ethnicity, and delayed treatment initiation are the main factors associated with the risk of renal relapses in a population-based study on SLE patients enrolled between 2000 and 2015 in the United States. The leading causes of death were the young age in women, (mainly between 15–24years) and the African-American and Hispanic origin (41).

## Standard treatment of severe lupus nephritis

Increased awareness of severe forms of LN can help to improve the treatment and outcome. According to the Joint EULAR/ERA-EDTA, patients with class III or IV LN and high activity index should start therapy with three intravenous (iv) methylprednisolone pulses (MPP) and an immunosuppressive agent, mycophenolate mofetil (MMF) or cyclophosphamide (CYC). After MPP, patients should receive oral prednisone at progressive lower dosage (5). The Aspreva Lupus Management Study (ALMS) demonstrated that 3 g of MMF and monthly iv CYC pulses have the same efficacy and similar side effects in Caucasians and in Asian patients while in “other ethnicities”, including African-Americans and Hispanics participants, MMF was significantly more effective than CYC (42). After these results, MMF became the drug of choice in LN patients with these ethnicities. However, in the ALMS, only half of patients achieved response at six months, defined as ≥50% reduction of proteinuria and stabilization of renal function (43).

For severe nephrotic syndrome, the recent EULAR/EDTA recommendations suggest the combination of glucocorticoids with 1-2 g/day of MMF and a calcineurin inhibitor (CNI), preferably tacrolimus [5]. This association of drugs, called multitarget therapy, demonstrated a significant higher rate of response at six months than high monthly dose of iv CYC, in a Chinese study (44). The efficacy of CNI in reducing proteinuria is well known and is due to two different mechanisms: the vasoconstriction of the afferent arteriolas leading to reduced GFR and reduced urine protein excretion, and the stabilization of podocyte cytoskeleton (45, 46). However, CNI are not easy to handle and may be responsible of important side effects including hypertension, diabetes, and nephrotoxicity.

The rare cases of AKI with normal glomeruli at light microscopy that can develop during lupus podocytopathy, usually respond well to corticosteroids and immunosuppressive agents but may show a high rate of relapses. Instead, in case of AKI and focal segmental glomerulosclerosis(FSGS) the response to therapy is worse with a low rate of complete remission (47). Collapsing variants of FSGS in LN, with poor response to aggressive therapy have been reported (48).

Until few years ago patients with Lupus TMA were treated with plasma infusions or plasmapheresis. This treatment reduced the mortality in comparison with patients who did not receive these treatments, but many patients did not respond to this therapy (49). Eculizumab, a recombinant, fully humanized IgG2/IgG4 monoclonal antibody that inhibits C5activation, proved to be a very efficacious therapy in lupus TMA (50).

## New treatments for severe lupus nephritis

New drugs have been tested to increase the rate of response, diminish the risks of flares, and reduce the doses and side effects of corticosteroids and CNI (**Table 1**).

**Table 1:** Results of recent randomized controlled trials in Lupus nephritis.

TRIAL	Criteria for response	Study drug	Placebo	Difference	Results
Results at 1 year					
LUNAR **Rituximab** (51)	UPCR <0.5, creat <15%, <5 RBC	30.6	26.4	4.2%	Failure
NOBILITY CRR **Obinotuzumab** (52)	UPCR <0.5, creat <15%, <10 RBC	34.9%	22.6%	12.3%	Failure
NOBILITY ORR **Obinotuzumab** (52)	UPCR <0.5 creat <15%,	40%	18%	22%	Success
AURA III **Voclosporin** (53)	UPCR<0.5 eGFR ≥60ml/min	40.8%	22.5%	18.5%	Success
BLISS-LN **Belimumab** (54)	UPCR<0.7, eGFR <20% and≥60ml/min	46.6%	35.4%	11%	Success

UPCR, urinary protein/creatinine ratio; creat, serum creatinine; RBC, red blood cells; eGFR, estimated glomerular filtration rate.

The long-term use of glucocorticoids may increase the risk of obesity, glucose intolerance, hypertension, dyslipidaemia, and other atherogenic factors, with consequent elevated cardiovascular morbidity and mortality (55–58). In addition, glucocorticoids are among the most important determinants of chronic damage development in LN (59–62). A multivariate analysis on 187 biopsy-proven LN patients followed for around 18 years showed that an average prednisone dosage>5mg/day evaluated along the whole follow-up was one of the independent predictors of the first increase in the Systemic Lupus International Collaborating Clinics American College of Rheumatology Damage index(LICC/ACR DI) (63). Belimumab, a recombinant human IgG-1λ monoclonal antibody that inhibits B-cell activating factor, can increase the rate of response in SLE, decrease the rate of flares, and reduce the corticosteroid dosage (64). In a phase III randomized controlled study, participants were assigned to receive standard therapy (corticosteroids and 3g MMF or low-dose ivCYC) plus placebo or standard therapy plus Belimumab 10mg/kg administered intravenously once a month for two years. At the end of the follow-up, more patients in the belimumab group than in the placebo group had a primary efficacy renal response (43%vs.32%;P=0.03) and a complete renal response (30%vs.20%;P=0.02). The results were even better when belimumab was associated with MMF in class III and in Class IV LN in comparison to mixed and membranous forms and in non-black patients. The risk of a renal-related events or death was lower with belimumab than placebo (hazard ratio, 0.51;P=0.001). At the last observation (104 months) a significantly higher number of patients in belimumab were in treatment with ≤5mg prednisone. No difference with placebo was reported in side effects (65). A secondary analysis of this study demonstrated that belimumab significantly reduced the risk of renal flares and attenuated the annual rate of estimated glomerular filtration rate (eGFR) decline (54). On the other hand, response was significantly more frequent when urine protein/creatinine ratio was <3g/g than when proteinuria was higher. These results suggest that adding belimumab to standard initial therapy in severe LN not only can improve the response but may also reduce renal flares and the corticosteroids dosage. Based on these results, belimumab in association with MMF can be added to induction therapy in active class IV LN, in patients with history of renal relapses and in those who require a reduction/withdrawal of corticosteroids.

Recently, in a phase III randomized controlled trial a new CNI, voclosporin, employed at the dosage of 23,7g twice a day, in association with 2g of MMF and glucocorticoids, demonstrated the superiority at six and at 12 months in comparison to placebo plus MMF and glucocorticoids in inducing complete or partial remission of LN. Of interest, these results were obtained with very low dosage of corticosteroids; 20-25mg/day of prednisone at the start of the study rapidly reduced to 2.5 mg/day at week 12. Instead, no changes from baseline in immunological parameters (serum levels of C3, C4, anti dsDNA antibodies) were observed. The results were less good in Caucasians than in other ethnicities and in membranous than proliferative LN. Serious side effects, mainly pneumonia, were similar in the two groups (66). Voclosporin is reported to be more potent than cyclosporin *in vitro*, with a more stable pharmacodynamic and pharmacokinetic that avoids the need of blood monitoring; the lipid and glucose metabolic profiles have been reported to be better than those observed with the old formula of cyclosporine (53). No change in GFR after 52 weeks was demonstrated in the two groups. However, patients with an eGFR ≤ 45mL/min/per1,73m² at screening were excluded from the study. The potential nephrotoxicity of CNI is well known. To verify the superiority of voclosporin over other CNIs in LN a randomized trial should compare the efficacy of voclosporin vs low- dose cyclosporine or tacrolimus. Long term data and the identification of eGFR cut-off for voclosporin contraindication are necessary (67, 68). Waiting for these results, the combination of CNI, and low dose MMF and low dose corticosteroids can be helpful in patients with active proliferative LN and severe proteinuria, with the aim to induce a rapid resolution of proteinuria particularly if kidney function is normal, good control of arterial hypertension, and low chronicity index at kidney biopsy (69). Similarly, voclosporin can be started in association with MMF and with low dose corticosteroids in active proliferative LN particularly in presence of severe nephrotic syndrome. Although it seems not necessary to check the blood levels of voclosporin, renal function monitoring is necessary particularly during the first months of therapy to modulate the dosage of the drug.

## Severe LN based on severe clinical/histological presentations

Although presentation with nephritic syndrome or with rapidly progressive renal insufficiency seem to have progressively reduced during the last forty years at least in developed countries (2), kidney dysfunction at presentation is an important predictor of CKD (70). Even if discrepancies between clinical and histological presentations exist, impaired kidney function at presentation is usually associated with the presence of cellular crescents, tuft fibrinoid necrosis and/or severe diffuse interstitial infiltrations at kidney biopsy (11, 71). In these cases, the complete response to therapy is difficult to achieve and renal prognosis is poor. In our personal experience of 213 biopsies proven LN patients followed for around 10 years, those with eGFR<60ml/min/per1,73 m² at time of renal biopsy developed a significantly higher rate of ESKD (17.6%) than those presenting with eGFR ≥60mil/min/per 1,73 m² (2.7%, P=0.001) **(** **Table 2** **).** Whether MMF and CYC are equally effective in treating the severe forms of LN is an open question. Before the advent of MMF, many units used monthly administration of high doses of iv CYC, as suggested by the results of randomized, controlled, trials in which a large percentage of patients with severe LN were included (72, 73). More recently, LN patients at high risk were excluded from clinical trials or were included in very low percentage. However, a sub analysis of the Aspreva study included patients with an eGFR<30 ml/min at randomization treated with either high-dose iv CYC (12 patients) or 3gMMF (20 patients). At the start of that study there were no significant differences in the clinical characteristics between the two groups. Scarring at kidney biopsy were present in 42% of patients in the CYC group and in 35% of MMF group. The difference was not significant but the score of chronicity index in the two groups was not reported, although chronicity index is one of the best predictors of kidney function deterioration in the long-term (12). The more rapid response in MMF group was probably due to more active lesions in MMF than in CYC participants. At six months, no difference in the response was demonstrated between the two groups, but only few patients responded to treatment 16.7% in the CYC arm vs 20% in MMF (74). A pooled analysis reported the effects of CYC or MMF in all the published cases with impaired kidney function at presentation and/or crescents in more than 15% of glomeruli and fibrinoid necrosis. The two drugs appeared to be equally effective in inducing remission in the short term. Among 139 participants to this analysis, the average partial remission (48%MMF;51%CYC) and complete remissions (9%MMF;6%CYC) at 6 months were similar (75). However, in the maintenance phase, relapse rates and risk of developing ESKD were higher for MMF than for CYC (75–77). Looking at those rates of response it seems that neither CYC nor MMF can successfully manage patients with severe LN. In our experience, the induction therapy of severe forms consisted of three intravenous MPP (500-1000 mg/die) followed by oral prednisone 0.75-1 mg/kg/die for 2-4 weeks tapered to 10 mg/day, associated with oral cyclophosphamide (1.5-2 mg/kg/die) for at most 3 months. We checked every 7-10 days the number of white blood cells and adjusted the dosage of cyclophosphamide accordingly. One important point is the regular monitoring of the patients particularly during the first months. If renal function did not recover within two/three months a new course of methylprednisolone pulses, or, more recently, a rituximab infusion of 1g was added. In case of worsen of renal function, a repeat kidney biopsy can be of help to exclude over-imposed TMA.

**Table 2:** Rate of ESKD and death in patients with Lupus Nephritis with eGFR < or ≥ 60/mil/min at kidney biopsy.

	eGFR <60ml/min/	eGFR≥60ml/min/	p
Patients	68	145	
eGFR ml/min	47.7 (28.1-50.4)	101 (79-122)	0.0001
Proteinuria g/day	4 (1.9-55)	2.9 (1.9-4.9)	0.30
Histologic classes II/III/IV/V	0/13/49/6	2/33/66/44	0.0002
Arterial hypertension	66.2%	42.1%	0.002
Activity index	8 (4.75-10)	4 (1-8)	0.0001
Chronicity index	2 (1-5)	1 (0-3)	0.0001
Follow-up years	10.7 (2.1-22.1)	11-8 (4.2-20.1)	0.91
ESKD	17.6%	2.7%	0.0001
Deaths	13.2%	4.1%	0.02

Personal experience. eGFR, estimated glomerular filtration rate; ESKD, end-stage kidney disease.

It is possible, and even likely, that an add-on therapy with a novel agent may be helpful, especially in presence of low chronicity index.

Many hopes rest on the efficacy of Rituximab (RTX) a chimeric monoclonal antibody directed against CD20. The Explorer trial failed to show the superiority of RTX over placebo in lupus patients treated with azathioprine, mycophenolate mofetil, and methotrexate (78) and the Lunar trial showed that RTX therapy did not improve clinical outcomes of LN after 1 year of treatment (79). However, it is possible that the inclusion of patients with mild-moderate LN can have influenced the results, since most patients assigned to the control arm obtained a high rate of response with standard therapy, so obscuring the potential superiority of RTX. Despite these negative results of randomized, controlled trials, RTX continues to be used with success in refractory or frequently relapsing forms of LN (51, 80–82). Cases of good response of the association of CYC and RTX in severe and refractory LN have also been reported in literature (83, 84). In a study, 84 patients with severe and refractory LN were randomized to CYC or to the association of CYC+RTX. The response occurred in 83.3% of participants assigned to the combined therapy and in 57.1% of those assigned to CYC alone, the difference being significant. Proteinuria, serum C3 fractions and SLEDAI score were significantly improved in RTX group (85).

Obinotuzumab, a humanized type II anti-CD20 monoclonal antibody, demonstrated to be superior to RTX in the treatment of follicular lymphoma (86). Recently, Obinotuzumab at the dosage of 1g at day 1, at week 2, 24, and 26 in association with methylprednisolone pulses and 2-2.5 g of MMF was compared with placebo in a phase II randomized trials on 125 patients with proliferative LN. The primary end-point, complete renal response, was not achieved at week 52, despite a percentage difference of 12% in favor of Obinotuzumab. Instead, in an exploratory analysis conducted at week 104, complete renal response was reported in a significantly higher number of patients in Obinotuzumab (41%) in comparison to placebo (23%,P=0.026) (87). If these mid-term results will be confirmed by further studies, one can hope that this monoclonal antibody in association with standard therapy may improve the current results in severe forms of LN. A phase III randomized trial are under way. Among other drugs in study, anirolumab a monoclonal antibody against type I interferon is ongoing (52), a Janus-kinase inhibitor suppressing signals from multiple cytokines, and secukinumab a selective inhibitors of interleukins-17 are under way (88).

## Conclusions

Despite progressive improvement in renal survival, lupus nephritis continues to be a disease with high risk of ESKD and death. The management of severe LN is a real challenge. It requires a careful clinical/histological assessment to predict the short-term and long-term outcomes, the choice of a treatment that may couple efficacy and safety, and a regular monitoring of patients by a dedicated team.

The simultaneous or alternate combination of time-honored and new promising drugs might allow to interfere with different pathogenetic mechanisms of the disease, achieve higher rate of complete and stable response, prevent flares, and reduce the dosage of corticosteroids. Further drugs for the treatment of LN are under investigation.

## Author contributions

GM contributed to conception and design of the review and wrote the first draft of the manuscript. All authors contributed to manuscript revision, read, and approved the submitted version.

## Conflict of interest

The authors declare that the research was conducted in the absence of any commercial or financial relationships that could be construed as a potential conflict of interest.

## Publisher’s note

All claims expressed in this article are solely those of the authors and do not necessarily represent those of their affiliated organizations, or those of the publisher, the editors and the reviewers. Any product that may be evaluated in this article, or claim that may be made by its manufacturer, is not guaranteed or endorsed by the publisher.
